# Development of an Immunochromatographic Strip for Rapid Detection of Canine Adenovirus

**DOI:** 10.3389/fmicb.2019.02882

**Published:** 2019-12-11

**Authors:** Shujie Wang, Yongjun Wen, Tongqing An, Guixin Duan, MingXia Sun, Jinying Ge, Xi Li, Kongbin Yang, Xuehui Cai

**Affiliations:** ^1^National Key Laboratory of Veterinary Biotechnology, Harbin Veterinary Research Institute, Chinese Academy of Agricultural Sciences, Harbin, China; ^2^College of Veterinary Medicine, Inner Mongolia Agricultural University, Hohhot, China; ^3^Department of Neurosurgery, The First Affiliated Hospital of Harbin Medical University, Harbin, China

**Keywords:** canine adenovirus, colloidal gold immunochromatographic strips, rapid detection, hexon protein, monoclonal antibodies

## Abstract

Although canine adenovirus (CAdV) is highly prevalent in dogs, there is currently a lack of a quick diagnostic method. In this study, we developed a rapid immunochromatographic strip (ICS) assay using colloidal gold coupled to CAdV-2-specific monoclonal antibodies (mAbs). BALB/c mice were immunized with a purified CAdV-2 suspension, and four mAbs (belonging to two different epitopes) were generated and designated as 2C1, 7D7, 10D1, and 4G1. Western blot and protein spectral analysis indicated that the hexon protein of CAdV-2 recognized all four mAbs. The colloidal gold-coupled 7D7 and 2C1 mAbs were chosen for inclusion in the rapid ICS assay. The optimal concentrations of the coating antibody (2C1), the capture antibody (7D7), and the goat anti-mouse antibody were 1.0 mg/ml, 10 μg/ml, and 2.0 mg/ml, respectively. The limit of detection was approximately 2.0 × 10^2^ tissue culture infective dose (TCID_50_)/ml. Other common canine viruses were tested to evaluate the specificity of the ICS, and positive results were observed for only CAdV-1 and CAdV-2. The ICS test was conducted on 360 samples to detect CAdV, and the results were compared with those of polymerase chain reaction (PCR) tests. The ICS test was found to be a sufficiently sensitive and specific detection method for the convenient and rapid detection of CAdV.

## Introduction

Canine adenovirus (CAdV; family *Adenoviridae*; genus *Mastadenovirus*) is a non-enveloped dsDNA virus that infects numerous mammalian carnivores (Morrison et al., 1997; Balboni et al., 2013). There are two sub-types: CAdV type 1 (CAdV-1) and CAdV type 2 (CAdV-2); CAdV-1 is the etiological agent of infectious canine hepatitis, whereas CAdV-2 causes infectious tracheobronchitis and canine enteritis in domestic dogs (Macartney et al., 1988; Almes et al., 2010). Clinical case reports of CAdV-1 (Headley et al., 2013; Balboni et al., 2017) and CAdV-2 (Hamelin et al., 1986; Spibey and Cavanagh, 1989; Buonavoglia and Martella, 2007) support the hypothesis that CAdV continues to circulate in domestic dogs.

CAdV-1 and CAdV-2 are also widespread in wildlife. CAdV-1 primarily causes subclinical infection, but can be epizootic in wild carnivores belonging to the Canidae, Mustelidae and Ursidae families (Hechinger et al., 2017; Dowgier et al., 2018; Balboni et al., 2019). CAdV-1 has been reported in different fox species in several geographic areas (Thompson et al., 2010; Walker et al., 2016a, b). Serological and pathogen surveys conducted in Thailand, Scandinavia, South Africa, Italy, and Turkey demonstrate the high prevalence of CAdV-2 infections in wild dogs and red foxes (Balboni et al., 2013; Bulut et al., 2013).

The current methods for diagnosing and identifying CAdV infection are usually based on serological tests (Hu et al., 2001), polymerase chain reaction (PCR) (Hu et al., 2001) and TaqMan real-time PCR (Hechinger et al., 2017). These methods are time-consuming, expensive, and mostly restricted to well-equipped laboratories. Therefore, developing a rapid detection method to monitor CAdV is desirable for field research and surveillance.

The colloidal gold-based immunochromatographic strip (ICS) assay is rapid and convenient to use, requires minimal equipment, and is widely used in some fields (Meng et al., 2014). However, no reports have been published regarding detection of CAdV using this type of assay. Here, we developed an ICS using colloidal gold coupled with CAdV-specific monoclonal antibodies (mAbs), allowing for rapid detection and surveillance with high sensitivity and specificity.

## Materials and Methods

### Animal Ethics Statements

This study was conducted in accordance with the recommendations in the Guide for the Care and Use of Laboratory Animals of the Ministry of Science and Technology of China. The Committee on the Ethics of Animal Experiments of the Harbin Veterinary Research Institute of the Chinese Academy of Agricultural Sciences reviewed and approved the protocols. CAdV-2 immunization experiments were conducted on 6-week-old BALB/c mice within the animal biosafety level 2 facilities of the Harbin Veterinary Research Institute of the Chinese Academy of Agricultural Sciences (approval number SY-2016-MI-013).

### Viruses and Reagents

The CAdV-2 strain used in this study was stored at the Harbin Veterinary Research Institute of the Chinese Academy of Agricultural Sciences. CAdV-2 was propagated in Madin-Darby canine kidney (MDCK) cells. CAdV-1, canine rabies virus (CRV), canine distemper virus (CDV), canine parvovirus (CPV), canine parainfluenza virus (CPIV), canine coronavirus (CCV), and canine leptospira virus (CLV) were collected at the Harbin Veterinary Research Institute of the Chinese Academy of Agricultural Sciences. Chlorauric acid was purchased from Harbin Chemical Reagents (Harbin, China). Nitrocellulose membranes, absorbent paper, sample pads, conjugate pads, and polyvinyl chloride (PVC) sheets were purchased from Millipore (Shanghai, China).

Polymerase chain reaction was used to specifically identify CAdV-2 (Hu et al., 2001) using the following primer sequences: forward 5′-CGC GCT GAA CAT TAC TAC CTT GTC-3′ and reverse 5′-CCT AGA GCA CTT CGT GTC CGC TT-3′. PCR products were sequenced by the HuaDa Gene company (Beijing, China).

### Preparation of Monoclonal Antibodies

Canine adenovirus 2 was propagated and purified, then used to produce mAbs as previously described (Huang et al., 2007; Yin et al., 2012). Briefly, five 6-week-old female BALB/c mice were immunized with 60 μg purified CAdV-2 emulsified with complete Freund’s adjuvant (Sigma-Aldrich). Two booster immunizations containing purified CAdV-2 and equal volume of incomplete Freund’s adjuvant (Sigma-Aldrich) were performed at 2-week intervals. Three days after the final booster, spleens were removed and splenocytes were fused with SP2/0 myeloma cells. Hybridoma cell lines secreting antibodies against CAdV-2 were screened and subcloned at least three times by limiting-dilution (Greene et al., 1980). Hybridoma culture supernatants were screened for antibodies using indirect ELISA. Antibodies that bound to the CAdV-2 virus but failed to bind MDCK cells were considered CAdV-2-positive. The stable cells were injected into the abdominal cavities of BALB/c mice, which had been pretreated with liquid paraffin. Approximately 1 week later, the mAbs were harvested and purified from the seroperitoneum using an antibody purification kit (HiTrap Protein G HP, GE Healthcare, Milwaukee, WI, United States) per the manufacturer’s instructions.

### Characterization of mAbs Against CAdV-2

After infecting the MDCK cells with CAdV-2 at a multiplicity of infection (MOI) of 10 and incubating them at 37°C for 24 h, the infected and uninfected cells were fixed with 4% paraformaldehyde for 15 min and probed with different mAb supernatants (SP2/0 supernatants as control) for 1 h at 37°C. Bound antibodies were visualized using fluorescent-conjugated antibodies against mouse IgG (TIANGEN, Beijing, China) on a fluorescence microscope.

Canine adenovirus 2 suspension samples (15 μl) were loaded on 12% (w/v) SDS-PAGE gels and transferred from the gels to polyvinylidene fluoride (PVDF) membranes (ISEQ00010, Millipore, Billerica, MA, United States). After blocking with 10% dried milk dissolved in phosphate-buffered saline (PBS) at 4°C overnight, the membranes were incubated with the mAb and SP2/0 supernatants for 1.5 h at room temperature. The membranes were then incubated with peroxidase-conjugated goat anti-mouse IgG (Sigma-Aldrich, St. Louis, MO, United States) for 1 h. Immunoreactive bands were visualized using an enhanced chemiluminescence system (ECL; PerkinElmer Life Sciences, Fremont, CA, United States). The protein bands that had reacted with the mAbs were subjected to MALDI-TOF-MS analysis. Mass spectra were acquired using an Ettan MALDI-TOF Pro mass spectrometer (GE Healthcare, Uppsala, Sweden) by Sensichip Infotech Co., Ltd. (Shanghai, China). The three-dimensional (3D) structure of identified protein was predicted by SWISS-MODEL online server and analyzed with PyMOL software.

### Identification of Different Epitopes via ELISA Additive Tests

An additive index (AI), which compared the optical densities (ODs) obtained for the two mAbs assayed under standardized conditions (either alone or in a mix) was calculated for each pair of mAbs (Friguet et al., 1983) using the formula, AI = {[2 × A_1__+__2_/(A1 + A2)] - 1} × 100, where A1 and A2 refer to the ODs obtained when the mAbs were assayed separately, and A_1__+__2_ refers to the OD obtained when the same amounts of the two mAbs were pooled in the same well. The mAb concentrations were assumed to be saturated for the purified virus. If both mAbs bound to the same epitope, the AI would be negligible; otherwise, the AI would be near 100 if the two epitopes were topographically unrelated. The lowest AI reported for the mAbs at different epitopes on CAdV-2 was used as the threshold for appraising epitopic correlations.

### Preparation of the Colloidal Gold-mAb Conjugate and Construction of the ICS

First, the optimal antibody concentrations for the test lines, control lines and conjugation with the colloidal gold solution were determined as previously described (Liu et al., 2012). Next, 25-nm diameter colloidal gold particles were prepared as previously described (Grabar et al., 1997). Briefly, 100 ml of 0.01% (w/v) HAuCl_4_ was dissolved in double-distilled water and boiled with rapid stirring. Next, 2 ml of 1% trisodium citrate was quickly added. After boiling for 10 min, the solution was stirred continuously for 15 min until cooled. The pH was adjusted to 8.2 using 1% potassium carbonate (w/v), then stored at 4°C in a dark-colored glass bottle. Next, 300 μl of the mAbs were mixed with 20 ml of colloidal gold solution and stirred rapidly for 30 min, then 2.5 ml of 10% (w/v) bovine serum albumin was added to inhibit excess reactivity of the gold colloid, and the mixture was stirred for 30 min. The blend was centrifuged at 6000 × *g* for 45 min at 4°C. The obtained conjugate pellet was resuspended and washed twice using 2 mM borax buffer (pH 9.0) containing 0.1% (w/v) polyethylene glycol before being resuspended in 1 ml of the same buffer. The size and shape of both the unconjugated and conjugated colloidal gold were analyzed using transmission electron microscopy measurements based on the standard method.

**FIGURE 1:**
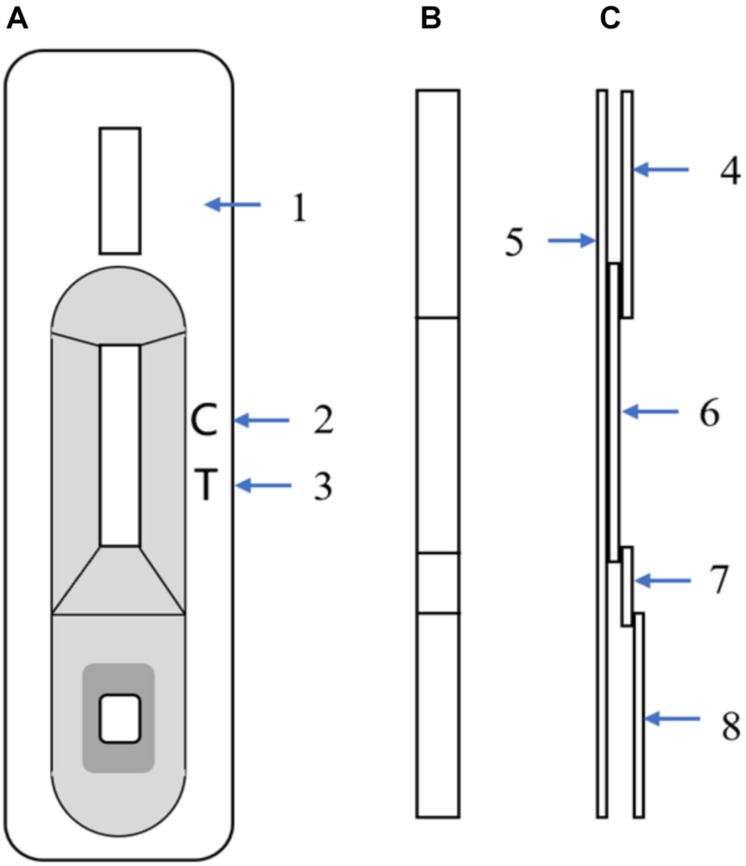
Schematic diagram of the immunochromatographic strip (ICS). **(A)** Front view of the ICS; (1) Plastic box, (2) Control-line position, (3) Test-line position (mAb 2C1, 1 mg/ml). **(B)** Strip front view. **(C)** Strip side view; (4) Absorbent paper, (5) PVC sheet, (6) Nitrocellulose membrane with control line and test line, (7) Glass-fiber membrane with mAb 7D7 (10 μg/ml), and (8) Glass-fiber membrane.

The ICS includes four components: an absorbent pad, a nitrocellulose membrane, a conjugate pad, and a sample pad. The nitrocellulose membrane was incubated with two antibodies: mAb 2C1 and goat anti-mouse IgG dissolved in PBS for the test and control lines, respectively. An XYZ3050 Dispense Workstation (BioDot, Inc., Sky Park, Irvine, CA, United States) was used to spray both antibodies, and the nitrocellulose membrane was then dried for an hour at 37°C before storing it at 4°C. The conjugate pad, composed of a glass-fiber membrane, was treated with a colloidal gold-mAb conjugate sprayed using an XYZ3050 Dispense Workstation at 10 μl/cm, then dried under a vacuum. All components, with some having been pretreated as described above, were adhered on a backing plate (300 × 70 mm) in the proper order. The plate was then cut into 4-mm-wide strips using a CM-4000 cutter (BioDot, Inc., Irvine, CA, United States). Figure 1 shows the schematic diagram of the ICS. The assembled strips were packaged in plastic boxes and stored at 4°C.

### Detection Principle and Test Procedure

In the testing process, liquid samples are dropped onto the sample pad, and a test line appears when samples contain CAdV-2. When the sample liquid reaches the conjugate pad, the CAdV-2 reacts with the colloidal gold-7D7 conjugate to form an antigen colloidal gold-7D7 complex. The complex then travels through the nitrocellulose membrane via capillary action. Finally, the complex reacts with mAb 2C1 on the test line, resulting in a dark red band. Conversely, in samples lacking CAdV-2, the superfluous conjugate or free conjugate that could not bind to the sample continues to travel to the control line. At the control line, the goat anti-mouse antibody reacts with mAb 7D7, and a dark red band appears. Therefore, within 10 min, two bands will appear for positive samples (one on the test line and one on the control line), whereas only one band will appear on the control line for negative samples.

### Specificity, Sensitivity, and Stability of the ICS

Common canine viruses were tested to evaluate the specificity of the ICS, including CAdV-1, CRV, CDV, CPV, CPIV, CCV, and CLV. CAdV-2 was used as the positive control; Dulbecco’s modified Eagle’s medium and MDCK cell culture supernatant were used as negative controls.

To evaluate the sensitivity of the ICS, 1.0 × 10^5.0^ tissue culture infective dose (TCID_50_)/ml CAdV-2 was serially diluted, either in sample dilution buffer (*Tris* hydrochloric acid system, pH 7.4) or negative dog serum, and 50 μl of each dilution was used for the ICS test. The sensitivity was determined by finding the minimum dilution concentration that yielded a positive result.

To ascertain the reproducibility and stability of the ICS during storage, 4 batches of strips (batch numbers: 180503, 180510, 180517, and 180524) were used to detect CAdV in sample dilution buffer (20 dog serum samples were chosen). All strips from each batch were stored at 4°C and used to test samples 4 times at 1-month intervals.

### Clinical Application of the ICS

Three hundred sixty clinical serum and rectal swabs (160 from a dog farm in Southern China and 200 from a dog farm in Northern China) were collected and tested for CAdV using the ICS test and PCR. Viral DNA was extracted from serum and rectal swabs using the DNA Extract Mini Kit (QIAGEN, Hilden, Germany) as per the manufacturer’s instructions. A portion of the E3 gene and its flanking regions was amplified using two conserved primers that produce different length fragments to differentiate between the two adenovirus types: 508 bp for CAdV-1 and 1030 bp for CAdV-2 (Hu et al., 2001; Chaturvedi et al., 2008). Thirty cycles were performed as follows: 95°C for 5 min, 94°C for 40 s, 58°C for 1 min, 72°C for 1 min, and 72°C for 10 min. The PCR products were electrophoresed on 1.2% agarose gel, and each experiment was repeated three times. The numerical data are expressed as the means ± SD and were analyzed using GraphPad Prism software (version 5.02 for Windows; GraphPad Software Inc., La Jolla, CA, United States). The difference between the ICS test and PCR results was assessed using one-way analysis of variance (ANOVA); *p* < 0.05 was considered statistically significant.

## Results

### Virus Identification

Canine adenovirus 2 from our institute was propagated and identified using specific primers by PCR. PCR products for the identified propagated virus were sequenced and compared by BLASTN to GenBank. The query sequence were identified as the E3 gene of CAdV-2, with 100% nucleotide identity to the subject sequence CAdV-2 isolate SV521/13 E3 gene (ID: KU725673.1).

### Characterization of mAbs to CAdV-2

**FIGURE 2:**
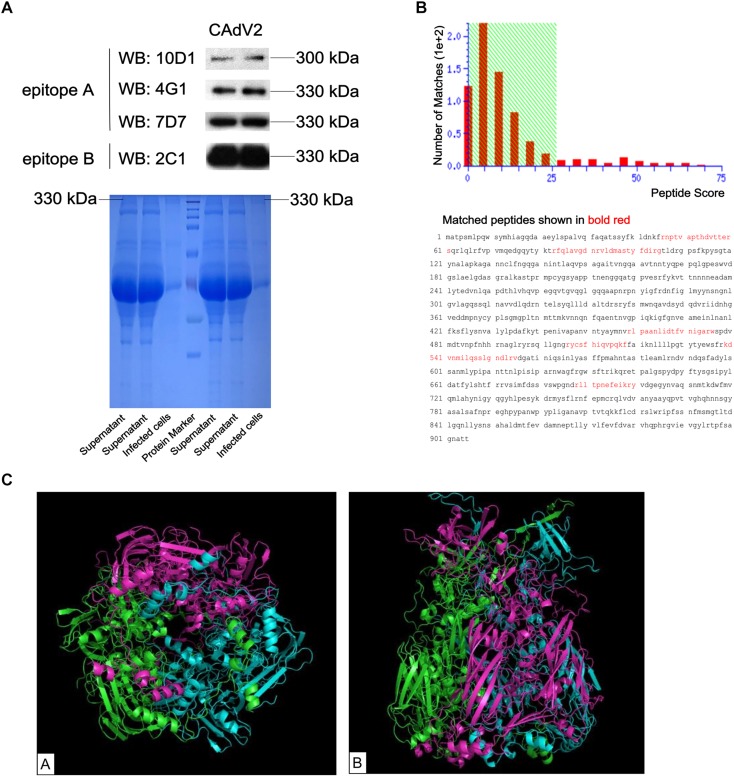
Identification of the CAdV-2 protein that reacted with mAbs. **(A)** Supernatant from CAdV-2-infected MDCK cells were subjected to western blot analysis using four mAbs (upper); Supernatant and infected cell lysates were subjected to SDS-PAGE (lower). **(B)** MALDI-TOF-MS results of the proteins identified in bands that reacted with the four mAbs. Protein scores that were greater than 26 were significant (*p* < 0.05). **(C)** Predicted 3D structure of the CAdV-2 hexon protein by the SWISS-MODEL online server; A, Bottom view of 3D structure; B, side view of 3D structure; different colors represent each monomer of the trimeric hexon protein.

The specificity of generated mAbs to CAdV-2 was determined using an indirect immunofluorescence assay (IFA) and western blot. IFA was performed on CAdV-2-infected MDCK cells to assess whether the mAbs recognized the wild type virus. All four mAbs reacted with the CAdV-2-infected MDCK cells, while no fluorescent signal was visualized in CAdV-2-uninfected MDCK cells and SP2/0 cells suspension (Supplementary Figure S1). Western blot results showed that these four mAbs specifically recognized the 330-kDa protein bands of CAdV-2 via SDS-PAGE (Figure 2A). Furthermore, the 330-kDa protein was analyzed via MALDI-TOF-MS on the basis of peptide mass matching. An NCBI database search revealed that the 330-kDa protein that reacted with all four mAbs was a hexon (69 peptides with scores larger than 26 were significant; *p* < 0.05; Figure 2B). Figure 2C shows the predicted 3D structure of the hexon protein with PyMOL software based on the predicted data from the SWISS-MODEL online server.

The ELISA additive test results revealed two distinct epitopes on CAdV-2: 2C1, 10D1 and 4G1 were mapped to epitope A, and 7D7 was identified as epitope B (Table 1). Of these, 7D7 and 2C1 reacted strongly with CAdV-2, and were selected for preparation of the ICS assay.

**TABLE 1:** Identification of different epitopes for four mAbs via ELISA additive tests.

**(AI)**			**10D1**	**4G1**	**2C1**	**7D7**
Epitope	A	10D1	–	–	–	100
Epitope	A	4G1	–	–	–	100
Epitope	A	2C1	–	–	–	100
Epitope	B	7D7	100	100	100	–

*AI = {[2 × A_1__+__2_/(A1 + A2)] - 1} × 100, where A1 and A2 refer to the ODs obtained when the mAbs were assayed separately, and A_1__+__2_ refers to the OD obtained when the same amounts of the two mAbs were pooled in the same well.*

### Optimization of ICS Antibody Concentrations

For the coating and control-line antibodies, purified mAb 2C1 and goat anti-mouse IgG were serially diluted, and the test-line and control-line colors gradually deepened as the mAb 2C1 and goat anti-mouse IgG concentrations increased (data not shown). Colorimetric reactions were optimal for a mAb (test-line) concentration of 1.0 mg/ml, and a goat anti-mouse IgG (control-line) concentration of 2.0 mg/ml; further increases above these concentrations did not significantly improve the sensitivity of detection.

**FIGURE 3:**
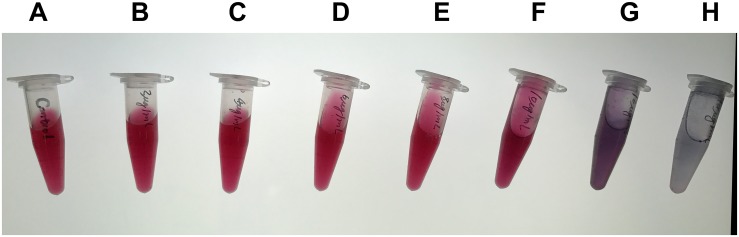
Optimization of the capture antibody concentration. Serial dilutions of purified capture antibody 7D7 were prepared containing 3, 4, 6, 8, 10, 12, and 14 μg/ml **(B–H)** and mixed with colloidal gold solution; colloidal gold solution alone served as a control **(A)**.

For the capture antibody, serial dilutions of purified mAb 7D7 were prepared containing 3, 4, 6, 8, 10, 12, and 14 μg/ml, and each dilution was separately added to the colloidal gold solution. As shown in Figure 3, the minimum concentration of mAb 7D7 needed to label with a stable wine red color was 8 μg/ml; therefore, an optimal concentration was chosen to be 10 μg/ml.

### Specificity of the ICS

**FIGURE 4:**
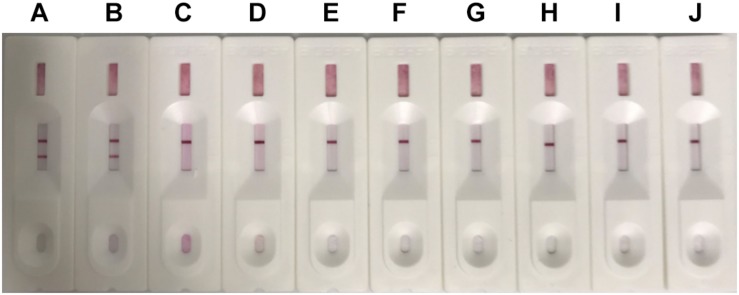
Specificity testing of the ICS. The following viruses were tested using the ICS assay developed in this study: canine adenovirus 1 (CAdV-1; strip **A**), CAdV-2 (strip **B**), canine rabies virus (CRV; strip **C**), canine distemper virus (CDV; strip **D**), canine coronavirus (CCV; strip **E**), canine parainfluenza virus (CPIV; strip **F**), canine leptospira virus (CLV; strip **G**), canine parvovirus (CPV; strip **H**), Dulbecco’s modified Eagle’s medium (control; strip **I**), and MDCK cells (control; strip **J**).

Different canine viruses (concentration > 10^4^ TCID_50_/ml) including CAdV-1, CDV, CRV, CPV, CPIV, CCV, CJEV, and CLV were collected to evaluate the specificity of the ICS assay (Supplementary Figure S2). Positive results were observed for both CAdV-1 and CAdV-2, while the other viruses yielded negative results (Figure 4). Thus, the ICS was highly specific for CAdV, and did not cross-react with the other pathogenic canine viruses.

### Sensitivity of the ICS

**FIGURE 5:**
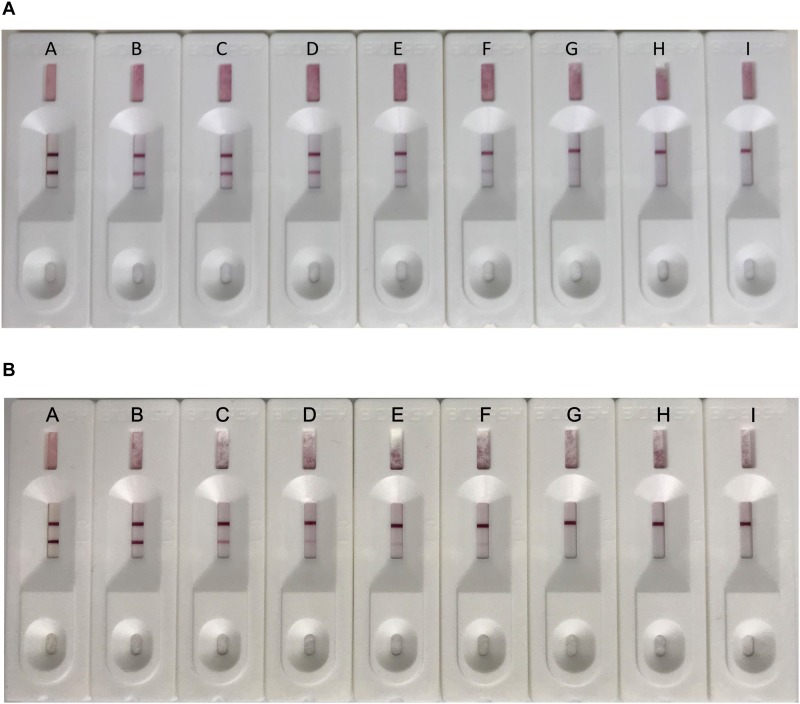
Sensitivity testing of the ICS. Serial dilutions of CAdV-2 were prepared in sample buffer **(A)** or negative dog serum **(B)** and tested using the ICS assay developed in this study. Dilutions of virus tested contained (A) 5 × 10^5^ TCID_50_/ml, (B) 5 × 10^4^ TCID_50_/ml, (C) 5 × 10^3^ TCID_50_/ml, (D) 2.5 × 10^3^ TCID_50_/ml, (E) 1.25 × 10^3^ TCID_50_/ml, (F) 1.0 × 10^3^ TCID_50_/ml, (G) 2 × 10^2^ TCID_50_/ml, (H) 10^2^ TCID_50_/ml, and (I) 10 TCID_50_/ml.

To determine the sensitivity of the ICS, serial dilutions of CAdV-2 were prepared in sample dilution buffer or negative dog serum and added to the ICS sample pad. The results showed that the limit of detection of the ICS was 2.0 × 10^2.0^ TCID_50_/ml (Figure 5A). The sensitivity assay was repeated 5 times, and all replicates were positive at 2.0 × 10^2.0^ TCID_50_/ml. However, the CAdV-2 diluted in dog serum reacted more slowly and color of the bands were lighter than in sample dilution buffer, the limit of detection of the ICS was 1.0 × 10^3.0^ TCID_50_/ml during 5 min (Figure 5B).

### Reproducibility and Stability of the ICS Assay

To determine its reproducibility and stability, the ICS assay was carried out with a different batch of strips after stored at 4°C 1, 2, 3, and 4 month. As shown in Table 2, 1 of the 20 tested serum samples was detected as positive and 19 of the 20 tested serum samples were detected as negative. Identical results can be seen for strips from the same batch and different batches after different times in storage.

**TABLE 2:** Test for reproducibility and stability of the ICS.

**Batch no.**	**180503**				**180510**				**180517**				**180524**			
				
**Times**	**1**	**2**	**3**	**4**	**1**	**2**	**3**	**4**	**1**	**2**	**3**	**4**	**1**	**2**	**3**	**4**
Positive no.	1	1	1	1	1	1	1	1	1	1	1	1	1	1	1	1
Negative no.	19	19	19	19	19	19	19	19	19	19	19	19	19	19	19	19

*Four batches of strips (batch numbers: 180503, 180510, 180517, and 180524) were used to detect CAdV in 20 dog serum samples diluted in buffer. All strips from each batch were used to test samples four times at 1-month intervals.*

### Clinical Application of the ICS

**FIGURE 6:**
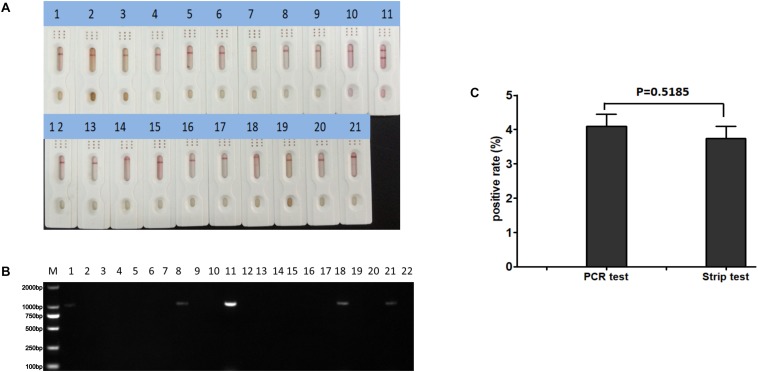
Practical application of the ICS assay. Detection of various clinical samples (1–10, 12–21) using **(A)** the ICS assay or **(B)** specific PCR. Positive (sample 11) and negative (sample 22) controls were included in each. **(C)** Rates of positive sample detection comparing the PCR and ICS test.

Polymerase chain reaction and the ICS were compared for their ability to detect CAdV in 360 canine clinical samples. The PCR detected 16 positive and 344 negative samples, whereas the ICS assay only detected 14 positive samples (Table 3). The positive rates of the PCR and ICS tests were 4.44 and 3.88%, respectively (Figure 6), indicating that the sensitivity of the ICS and PCR tests did not significantly differ (*p* > 0.05).

**TABLE 3:** Canine adenovirus detection results via ICS and PCR for clinical samples from China.

**PCR test**	**ICS test**		**Total**
		
	**Positive**	**negative**	
Positive	14	2	16
Negative	0	344	344
Total	14	346	360

## Discussion

The mAbs constructed in this study successfully recognized native-form CAdV-2. Western blot and MALDI-TOF-MS analysis revealed that all four mAbs recognized the CAdV-2 hexon protein. The trimeric hexon protein is the major structural component of the CAdV capsid, which appears relatively well conserved. The BLAST result showed that the CAdV-2 hexon protein had > 96% nucleotide identity and 100% coverage of the CAdV-1 hexon protein sequence, suggesting that the mAbs that recognized CAdV-2 may also recognize CAdV-1. Indeed, specificity testing showed that both could be detected by the ICS. The fact that the ICS is unable to distinguish between CAdV-2 and CAdV-1 might be an important limitation of this assay. Therefore, this ICS can serve as a quick field test that is used to direct which confirmatory PCR should be used to determine the type and help plan treatment. Moreover, there is a commercial ICS (Genbody, Korea) for rapid detection of CAdV-2 antigens on the market, so we can combine commercial ICS for CAdV-2 with our ICS for CAdV to determine the type. Of course, ICS testing must be also combined with a review of clinical symptoms of diseased animals in order to properly diagnose and treat in a clinical setting.

Virus isolation and real-time PCR assays are reliable methods for diagnosis of CAdV infections; however, these methods are mostly restricted to well-equipped laboratories. The ICS method has been widely applied to detect various infectious agents (Li et al., 2011; Nakayama et al., 2014; Liu et al., 2019), including canine viruses such as rabies and CPV (Tao and Li, 2014; Sharma et al., 2018). However, there are no publications regarding ICS detection of CAdV, although there is commercial ICS that detect CAdV-2 on the market. Therefore, we developed an ICS using colloidal gold coupled with two CAdV-2-specific mAbs, and it was highly specific for detection of CAdV-2 and CAdV-1. This novel ICS will be useful for rapid diagnosis of CAdV without the need for expensive equipment.

Among the main factors affecting the ICS specificity and sensitivity, the most important factor is mAb affinity (i.e., the mAb used on the test-line and the colloidal gold conjugate must have high affinity for the CAdV epitopes). In this study, we chose two mAbs which targeted different epitopes as capture and detector antibodies in order to guarantee the sensitivity and specificity of the ICS and prevent other possible antigens from conjugating to the antibody. The limit of detection of the test strip was 2.0 × 10^2^ TCID_5__0_/ml, which is more sensitive than the ICS developed using a single polyclonal antibody (pAb) and single mAb (limit of detection = 6.6 × 10^5^ TCID_50_/ml) (Sharma et al., 2018) or another using two mAbs (limit of detection = 2.9 × 10^3^ TCID_50_/ml) (Li et al., 2011). Its sensitivity was also higher than that of the commercial CAdV-2 test strip from Korean GenBody (limit of detection = 3.0 × 10^2^ TCID_50_/ml). In addition, the sample diluent is also an important part in the colloidal gold test strip system, as there is a risk of non-specific release without using the sample diluent. In our sensitivity testing, CAdV-2 diluted in serum took a longer time for the bands to appear, and the bands were lighter than in samples diluted in sample dilution buffer. Therefore, the samples must be properly diluted in the sample diluent during clinical testing.

In conclusion, we developed an ICS that could detect CAdV antigens with high specificity, reproducibility and stability. The ICS is able to detect CAdV in samples in approximately 5 min, which is much faster than PCR assays. In summary, our results suggest that this method may potentially be applied to initial clinical diagnosis of CAdV infection.

## Conclusion

This ICS is highly suited for the detection of CAdV in the field and has important clinical applications for diagnosis of this important canine pathogen.

## Data Availability Statement

All datasets generated for this study are included in the article/Supplementary Material.

## Ethics Statement

The animal study was reviewed and approved by the Committee on the Ethics of Animal Experiments of the Harbin Veterinary Research Institute of the Chinese Academy of Agricultural Sciences. Written informed consent was obtained from the owners for the participation of their animals in this study.

## Author Contributions

XC and KY conceived the study and designed the experimental procedures. SW, YW, GD, and XL performed the experiments. YW, GD, MS, and JG acquired the data. SW, YW, TA, and XL analyzed the data. SW, MS, XL, and JG contributed reagents and materials. SW, TA, KY, and XC wrote the manuscript. SW, TA, GD, MS, KY, and XC approved the version to be published.

## Conflict of Interest

The authors declare that the research was conducted in the absence of any commercial or financial relationships that could be construed as a potential conflict of interest.

## Supplementary Material

FIGURE S1 Canine adenovirus 2 detection using mAbs via indirect immunofluorescence assay. CAdV-2-infected MDCK and CAdV-2-uninfected MDCK were fixed in paraformaldehyde prior to immunostaining with specific anti-CAdV-2 mAbs: mAb 10D1; mAb 4G1; mAb 2C1; mAb 7D7; sp2/0 supernatant; scale bars: 75 μm.

FIGURE S2 Canine virus used for specificity testing of the ICS. The following viruses were tested using the ICS assay developed in this study: canine adenovirus 1 (CAdV-1; **A**), CAdV-2 **(B)**, canine rabies virus (CRV; **C**), canine distemper virus (CDV; **D**), canine coronavirus (CCV; **E**), canine parainfluenza virus (CPIV; **F**), canine leptospira virus (CLV; **G**), canine parvovirus (CPV; **H**), Dulbecco’s modified Eagle’s medium (control; **I**) and MDCK cells (control; **J**).
