# Supplementary figures and images for: Development of an Immunochromatographic Strip for Rapid Detection of Canine Adenovirus
Source: Front Microbiol. 2019 Dec 11;10:2882. doi: 10.3389/fmicb.2019.02882 (PMC6917642; doi:10.3389/fmicb.2019.02882)

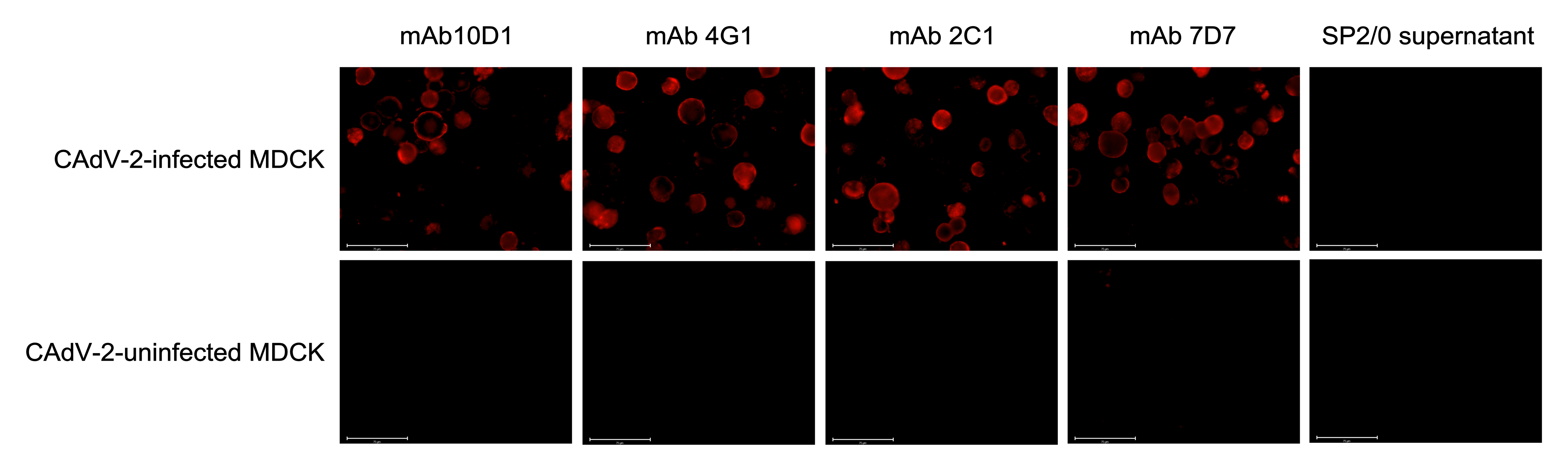

Supplement: FIGURE S1 — Canine adenovirus 2 detection using mAbs via indirect immunofluorescence assay. CAdV-2-infected MDCK and CAdV-2-uninfected MDCK were fixed in paraformaldehyde prior to immunostaining with specific anti-CAdV-2 mAbs: mAb 10D1; mAb 4G1; mAb 2C1; mAb 7D7; sp2/0 supernatant; scale bars: 75 μm. [file Image_1.tif]

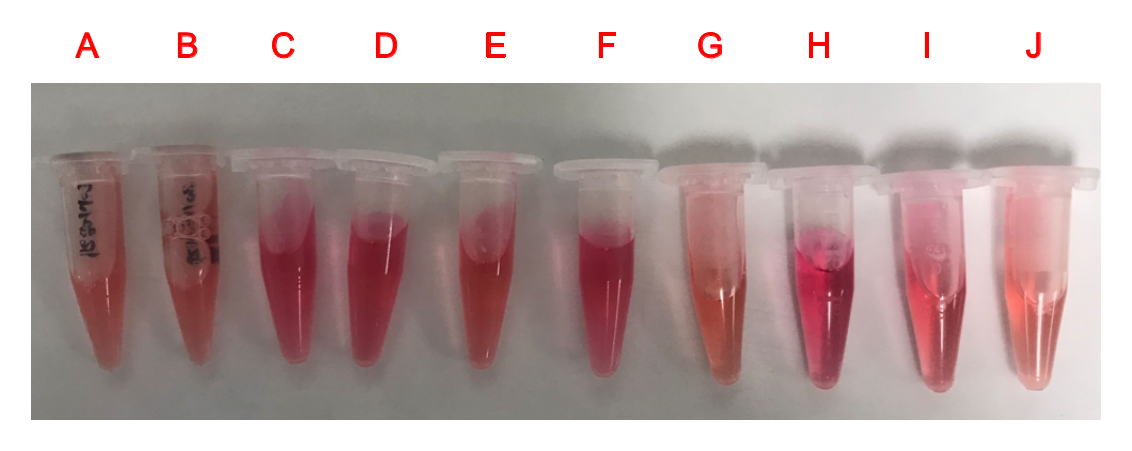

Supplement: FIGURE S2 — Canine virus used for specificity testing of the ICS. The following viruses were tested using the ICS assay developed in this study: canine adenovirus 1 (CAdV-1; A), CAdV-2 (B), canine rabies virus (CRV; C), canine distemper virus (CDV; D), canine coronavirus (CCV; E), canine parainfluenza virus (CPIV; F), canine leptospira virus (CLV; G), canine parvovirus (CPV; H), Dulbecco’s modified Eagle’s medium (control; I) and MDCK cells (control; J). [file Image_2.tif]
